# Sources for developing new medicinal products: biochemical investigations on alcoholic extracts obtained from aerial parts of some Romanian Amaryllidaceae species

**DOI:** 10.1186/s12906-018-2292-8

**Published:** 2018-07-27

**Authors:** Daniela Benedec, Ilioara Oniga, Daniela Hanganu, Ana Maria Gheldiu, Cristina Pușcaș, Radu Silaghi-Dumitrescu, Mihaela Duma, Brîndușa Tiperciuc, Rodica Vârban, Laurian Vlase

**Affiliations:** 1 Department of Pharmacognosy, “Iuliu Haţieganu” University of Medicine and Pharmacy, 12 I. Creangă Street, 400010 Cluj-Napoca, Romania; 2 Department of Pharmaceutical Technology and Biopharmaceutics, “Iuliu Haţieganu” University of Medicine and Pharmacy, 12 I. Creanga Street, 400010 Cluj-Napoca, Romania; 3 Department of Chemistry and Chemical Engineering “Babeş-Bolyai” University, 11 A. Janos Street, 400028 Cluj-Napoca, Romania; 4 State Veterinary Laboratory for Animal Health and Safety, 1 Piata Marasti Street, 400609 Cluj-Napoca, Romania; 5 Department of Pharmaceutical Chemistry, “Iuliu Hațieganu” University of Medicine and Pharmacy, 41 V. Babeş Street, 400012 Cluj-Napoca, Romania; 6 University of Agricultural Sciences and Veterinary Medicine, 3-5 Mănăştur Street, 400372 Cluj-Napoca, Romania

**Keywords:** *Galanthus*, *Leucojum*, *Narcissus*, Antioxidant and antimicrobial activities, Polyphenols, cytochrome *c*

## Abstract

**Background:**

Although *Galanthus nivalis* L. (snowdrop) is known for the galanthamine content, used in the treatment of Alzheimer disease, the polyphenolic compounds of Amaryllidaceae species are less studied. Proper understanding of the polyphenolics in these extracts and of their antioxidant and antimicrobial properties may allow a reconsideration of their medicinal uses.

**Methods:**

The polyphenolic content of four selected Amaryllidaceae species harvested from Romania (*Galanthus nivalis* L., *Narcissus pseudonarcissus* L., *N. poeticus* L. and *Leucojum vernum* L.) was determined by spectrophotometric methods; the identification of phenolic compounds was performed by a HPLC-MS method, in order to establish their polyphenolic fingerprints. For the evaluation of the antioxidant potential the following methods were employed: DPPH radical scavenging, FRAP, hemoglobin ascorbate peroxidase activity inhibition (HAPX), inhibition of lipid peroxidation catalyzed by cytochrome *c*, and electron paramagnetic resonance (EPR) spectroscopy assays. Antimicrobial activity was assessed using the disc diffusion method.

**Results:**

Qualitative and quantitative analyses highlight important amount of polyphenols (over 15 mg/g); the main identified compounds are chlorogenic and *p*-coumaric acids in all species. Only *G. nivalis* shows antioxidant activity by all the used methods. *G. nivalis* and *L. vernum* strongly inhibits the growth of *S. aureus*, while *N. poeticus* shows a very good antifungal activity.

**Conclusions:**

The results of this study provide a new approach to the properties and therapeutic uses of some Romanian widespread Amaryllidaceae species that could be considered sources of developing new medicinal products with anti anti-staphylococcal and antifungal activity.

## Background

The Amaryllidaceae family includes more than 1000 perennial bulbous species, distributed throughout the tropical and subtropical regions of the world. In the Romanian flora, about 17 species from five genera of this family can be found, spontaneous or cultivated. The most common Amaryllidaceae species are *Narcissus* sp. (daffodils), *Amaryllis* sp. and *Galanthus* sp. (snowdrops), mostly known as ornamental plants [1–3]. The Amaryllidaceae have been used for thousands of years as herbal remedies; thus, in the fourth century B.C. Hippocrates was using oil extracted from daffodil (*N. poeticus*) for the treatment of uterine tumors. Other species used in traditional medicine are *G. woronowii* and *G. nivalis,* indicated for the treatment of nervous system disorders, fainting, headache, heart and mitral insufficiency, myocarditis [3–5]. Over the past three decades, many specific alkaloids have been isolated (galanthamine, lycorine, crinine, haemanthamine, tazettine, narciclasine etc.) and reported to have acetylcholinesterase inhibitory effect (for the treatment of Alzheimer’s disease), antiviral, antibacterial, antifungal, antimalarial, antitumor and cytotoxic activities [3, 6–10]. In the aerial parts of some Amaryllidaceae flavonoids (hyperoside, quercetin, isorhamnetin-*O-*sophoroside, quercetin-3-*O*-sophoroside) and hydroxycinnamic acids (cinnamic, *p*-coumaric, *p*-hydroxybenzoic, caffeic acids) were identified [4, 5, 11–14]. The potential utility of these species is not just restricted to alkaloids, because the Amaryllidaceae synthesize a diverse array of polyphenols with well-known pharmacological properties (antioxidant, antimicrobial, anticancer, anti-aging etc.). Recent studies have shown the efficacy of natural polyphenols in Alzheimer’s disease through various mechanisms (antioxidant, acetylcholinesterase and butyrylcholinesterase inhibition). Also, chlorogenic acid is reported to have neuroprotective properties [15–18]. In this context, the aim of the present research was to determine the polyphenolic composition of some Romanian Amaryllidaceae species (*G. nivalis, N. pseudonarcissus, N. poeticus* and *L. vernum*) and to highlight some of their biological activities in order to reconsider the uses of these medicinal plants.

## Methods

### Plant materials

The aerial parts of 4 Amaryllidaceae species were harvested from different regions of Cluj County, Romania, in March 2017, during the flowering period. The plant materials were identified by PhD Rodica Varban (University of Agricultural Sciences and Veterinary Medicine, Cluj-Napoca) and PhD Ilioara Oniga (“Iuliu Haţieganu” University of Medicine and Pharmacy Cluj-Napoca). Voucher specimens of the 4 species are deposited at the Department of Pharmacognosy (“Iuliu Haţieganu” University of Medicine and Pharmacy Cluj-Napoca): *Galanthus nivalis* L. (Voucher No. 50), *Narcissus poeticus* L. (Voucher No. 81)*, N. pseudonarcissus* L. (Voucher No. 92)*,* and *Leucojum vernum* L. (Voucher No. 73).

### Chemicals and microorganisms

HPLC grade methanol and analytical grade orthophosphoric acid were purchased from Merck (Darmstadt, Germany). All chemicals and reagents were of analytical grade. Phenolic standards: chlorogenic, caffeic acid, *p*-coumaric acids, rutin, isoquercitrin, quercitrin, hyperoside, myricetol, fisetin, quercetin, apigenin, kaempferol were acquired from Sigma (St. Louis, MO, USA), ferulic, sinapic, gentisic, gallic acids, patuletin, luteolin were purchased from Roth (Karlsruhe, Germany), cichoric, caftaric acid were from Dalton (Toronto, ON, Canada). Other reagents or chemicals, including hydrochloric acid, aluminum chloride, sodium acetate, ethanol and Folin-Ciocalteu reagent were acquired from Sigma (St. Louis, MO, USA), sodium carbonate, hydrogen peroxide, sodium ascorbate and bovine hemoglobin were purchased from Sigma-Aldrich (Steinheim, Germany), DPPH (2,2-diphenyl-1-picrylhydrazyl), 2,4,6-tri(2-pyridyl)-1,3,5-triazine (TPTZ), 6-hydroxy-2,5,7,8-tetramethyl-chroman-2-carboxylic acid (*Trolox) were* obtained from Alfa-Aesar (Karlsruhe, Germany). All microorganism products were distributed by MicroBioLogics®: *Staphylococcus aureus* ATCC 6538P (Gram-positive bacteria), *Listeria monocytogenes* ATCC 13932 (Gram-positive bacteria), *Escherichia coli* ATCC 25922 (Gram-negative bacteria), *Salmonella typhimurium* ATCC 13076 (Gram-negative bacteria) and fungal strains *Candida albicans* ATCC 10231 and *Aspergillus brasiliensis* ATCC 16404. All spectrophotometric data were acquired using a Jasco V-530 UV-vis spectrophotometer (Jasco International Co., Ltd., Tokyo, Japan).

### Preparation of sample solutions

The powdered vegetal product (10 g) was extracted with 20 mL of 70% ethanol, in an ultrasonic bath (Polsonic Palczyski Sp. J., Poland, Sonic 3), at 60 °C and sonicated for 30 min. The extracts were filtered through paper filters in 20 mL flasks and then centrifuged at 4500 rpm for 20 min.; the supernatants were recovered [15–19].

### HPLC chromatographic conditions and instrumentation

Analysis was performed using an Agilent 1100 HPLC Series system (Agilent, Santa Clara, CA, USA) equipped with: G1322A degasser, G13311A binary gradient pump, column thermostat, G1313A autosampler, and G1316A UV detector coupled to an Agilent 1100 mass spectrometer. HPLC-MS analysis of the studied extracts was performed according to a previously validated and described method [15–19]. Separation of the compounds was carried out on a reverse-phase analytical column (Zorbax SB-C18 100 × 3.0 mm i.d., 3.5 μm particle); the column temperature was 48^0^ C. The detection was performed simultaneously using both the UV and the MS mode. The UV detector was set at 330 nm until 17.5 min, then at 370 nm. The MS system entailed an electrospray ion source in negative mode. The UV mode was employed for quantification after qualitatively positive identification by MS. The chromatographic data were processed using ChemStation and DataAnalysis software from Agilent. The mobile phase with methanol and acetic acid 0.1% (*v*/v) was used in a binary gradient. For 35 min, elution was performed with a linear gradient, starting at 5% methanol and finishing at 42% methanol. The flow rate of the mobile phase was 1 mL/min. Five microliters were used for injection. The standard MS spectra were integrated in a mass spectra library. Under these chromatographic conditions two couples of compounds (namely: caftaric vs. gentisic acid and caffeic vs. chlorogenic acid) could not be quantitatively determined due to peak overlappingș instead, these 4 carboxylic acids were determined only based on MS spectra, whereas for the rest of the compounds the linearity of the calibration curves was very good (R^2^ > 0.998), with detection limits in the range of 18 to 92 ng/mL. The detection limits were calculated as the minimal concentration yielding a reproducible peak with a signal-to-noise ratio greater than three. Analyses were performed using an external standard method; retention times were determined with a standard deviation ranging from 0.04 min to 0.19 min. The accuracy was between 94.13 and 105.3%, for all substances. In samples, the compounds were identified by comparison of their retention times and recorded electrospray mass spectra with those of standards recorded under the same conditions.

### Determination of the total polyphenolic and flavonoidic contents

The total polyphenolic content was determined according to the European Pharmacopoeia, using the Folin-Ciocalteu method, with a calibration curve of gallic acid (R^2^ = 0.999) and the results expressed as mg of gallic acid equivalent (GAE) per g dry weight (d.w.) [15–23]. A spectrophotometric method, based on flavonoid-aluminum chloride (AlCl_3_) complexation was employed for determination of the total flavonoidic content [15–19, 24]. In brief, 5 mL extract was mixed with 5.0 mL of sodium acetate 100 g/L, 3.0 mL of aluminum chloride 25 g/L, and filled up to 25 mL by methanol in a calibrated flask. The mixture was allowed to stand for 15 min. and absorbance was measured at 430 nm. The total flavonoidic content was calculated from a calibration curve (R^2^ = 0.999) and the result was expressed as mg rutin equivalent (RE) per g dry weight [15–19, 24].

### Determination of antioxidant properties

#### DPPH (2,2-diphenyl-1-picrylhydrazyl) assay

The antioxidant activity was determined by the 2,2-diphenyl-1-picrylhydrazyl (DPPH) assay, as described earlier, with some modifications. Briefly, 2.0 mL of methanolic DPPH solution (0.25 mM) were added to 2.0 mL of extract solution (or standard) in ethanol at different concentrations (18.75–150 μg/mL). After 30 min of incubation at 40 °C in a thermostated bath, the decrease in the absorbance was measured at 517 nm. The percent DPPH scavenging ability was calculated as: DPPH scavenging ability = (A_control_–A_extract_)/A_control_ × 100, where A_control_ is the absorbance of the DPPH radical and methanol (containing all reagents except the sample) and A_extract_ is the absorbance of the mixture of DPPH radical and sample extract. Trolox was used as a positive control [15–22, 25].

#### Ferric reducing antioxidant power (FRAP) assay

The FRAP (ferric reducing antioxidant power) method relies on the change in the color of a ferric complex of the 2,4,6-tri(2-pyridyl)-1,3,5-triazine (TPTZ) radical due to reduction of the metal to the ferrous form (Fe^+ 2^). Trolox was used for the calibration curve(correlation coefficient - 0.992) andthe results were converted to μM Trolox equivalents/100 mL extract [26, 27].

#### HAPX assay

The hemoglobin ascorbate peroxidase activity assay (HAPX) was previously described in detail [21, 28–31]. 5 μL extracts were added to ascorbate (120 μM) and peroxide (700 μM), in acetate buffer, pH 5.5. The reaction was triggered by met hemoglobin (6 μM) and it was monitored at 405 nm, where the changes are due to hemoglobin transformation. The antioxidant capacity is correlated with an increase in the inhibition time and is given in percent of inhibition [28–31].

#### Inhibition of lipid peroxidation catalyzed by cytochrome *c*

The inhibition of lipid peroxidation catalyzed by cytochrome *c* was performed as previously described [31]. Thus, liposomes were obtained from 5 mg/mL soybean lecithin suspended in phosphate buffer (20 mM, pH 7) and sonicated for 20 min in an ultrasonic bath (using a Power Sonic 410 device). The experiment monitored the formation of lipid conjugated dienes at 235 nm, at room temperature, in the presence of cytochrome *c* (2 μM) and extract (diluted 2 thousand times).

#### Direct detection of free radicals

For direct detection of free radicals [18, 29, 30], the extracts were diluted 25 times in ethanol 90% and treated with 5 mM NaOH (yielding a basic pH). The concentration of pure compounds was 2 mM in 90% ethanol. The measurements were performed very fast in a capillary tube placed in a holder of a Bruker ELEXYS E-580 spectrometer with continuous wave at X band (~ 9.4 GHz, modulation amplitude, 1 G, microwave power, 9.6 mM, center field 3514 and sweep field 100 G.

#### Determination of antimicrobial activity

The agar disk-diffusion assay is an official method used in many microbiology laboratories for antimicrobial susceptibility using specific culture media, various incubation conditions and interpretive criteria for inhibition zone [18, 32]. In this well-known procedure, agar plates are inoculated with a standardized inoculum of the test microorganisms***:*** *S. typhimurium, E. coli, L. monocytogenes, S. aureus, C. albicans,* and *A. brasiliensis*. After the hydration of the lyophilized strain, the sterile tampon was impregnated with hydrated material and transferred onto the selective medium specific for each strain (e.g *Salmonella*: Rambach agar, XLD agar; *E. coli*: TBX agar). The tampon was rotated with pressure and a circular area was inoculated on the agar media. Using a sterile loop, streaks were made repeatedly in the inoculated area and then streaked also on the rest of the plate’s surface. Immediately afterwards, the culture medium inoculated was incubated at corresponding temperatures (e.g. *Salmonella* 37 °C; *E. coli* 44 °C). From the pure ATCC reference culture, of 24 h, a 0.5 McFarland suspension was obtained (corresponding to 10^8 CFU/mL). The Muller-Hinton agar plates were inoculated by inundation. The plates were then dried in the thermostat for 20 min (this interval is not exceeded because the bacteria might reach a multiplication phase). The sterile disks were soaked with the tested solutions (50 μL of each extract). The plates were incubated overnight at 37 °C. The antimicrobial agent diffuses into the agar and inhibits germination and growth of the test microorganism. The diameters of inhibition growth zones were measured**.** Gentamicin, fluconazole and amphotericin B were used as *standard* antibacterial and antifungal *drugs.* The negative control was 70% ethanol (diameter = 6 mm). The clear halos greater than 10 mm were considered as positive results. Tests were performed in triplicate and values are the averages of three replicates.

The minimum inhibitory concentrations (MICs) of the extracts were determined by an agar dilution method including the same strains of microorganisms as used in the agar disk diffusion method [33]. For this experiment, 100 μL nutrient broths were placed in a 96 well plate and 100 μL of each plant extract were added in the first ten lines. Then 100 μL were aspirated from every well and placed in the second well line of the plate. This technique was used in order to obtain the desired dilutions until line 10; from the last well, 100 μL mixes were discharged as follows: 50.0 μL, 25.0, 12.5, 6.25, 3.12, 1.56, 0.78, 0.39, 0.19, and 0.09 μL of plant extracts in 100 μL medium. Each well was seeded with 5.0 μL of a 24 h culture bacterial suspension, adjusted to be similar to 0.5 McFarland scale 10^8 CFU/mL), and incubated for 16–24 h (48 h for fungi) at 35° ± 2 °C. MIC was detected by the lowest concentration of the analyzed product in which the development of the bacterium strain was inhibited (medium remained clear). Negative control (70% ethanol) concentration for the determination of MIC using serial dilution method, was similar with the concentration used in the 1st well of the plate [33].

#### Statistical analysis

The samples were analyzed in triplicate or more; the average and the relative SD were calculated using the Excel software package. The experimental data was evaluated using one-way analysis of variance (ANOVA), with *p* <  0.05 as threshold value for statistical significance. The statistical results confirm the hypothesis that the differences between the results are either not significant (*p* > 0.05), significant (0.001 < *p* <  0.05) or highly significant (*p* <  0.001).

## Results

### Qualitative and quantitative polyphenolic profile

**Fig. 1:**
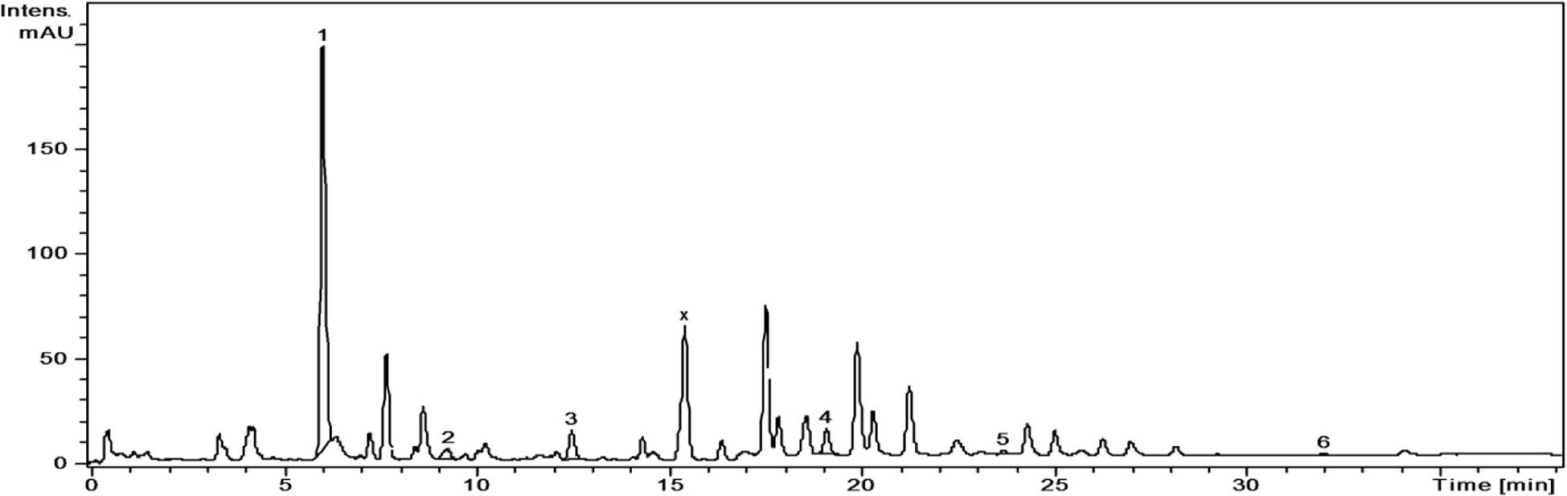
HPLC chromatograms of *N. pseudonarcissus* extract. *Notes:* The identified compounds: 1-chlorogenic acid; 2-*p*-coumaric acid; 3-ferulic acid; 4-hyperoside; 5-quercitrin; 6-kaempferol

**Fig. 2:**
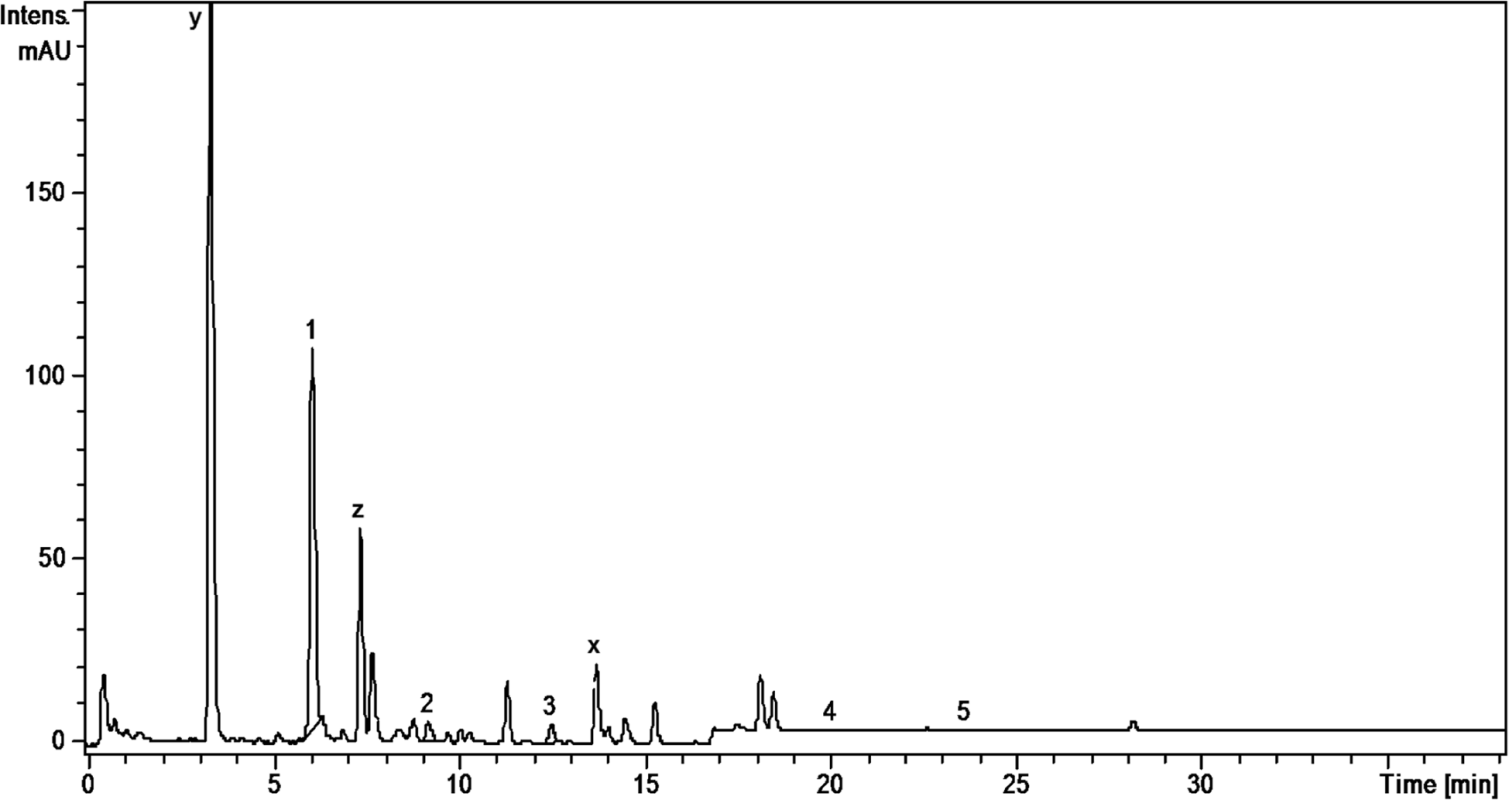
HPLC chromatograms of *N. poeticus* extract. *Notes:* 1-chlorogenic acid; 2- *p*-coumaric acid; 3-ferulic acid; 4-isoquercitrin; 5-quercitrin

**Fig. 3:**
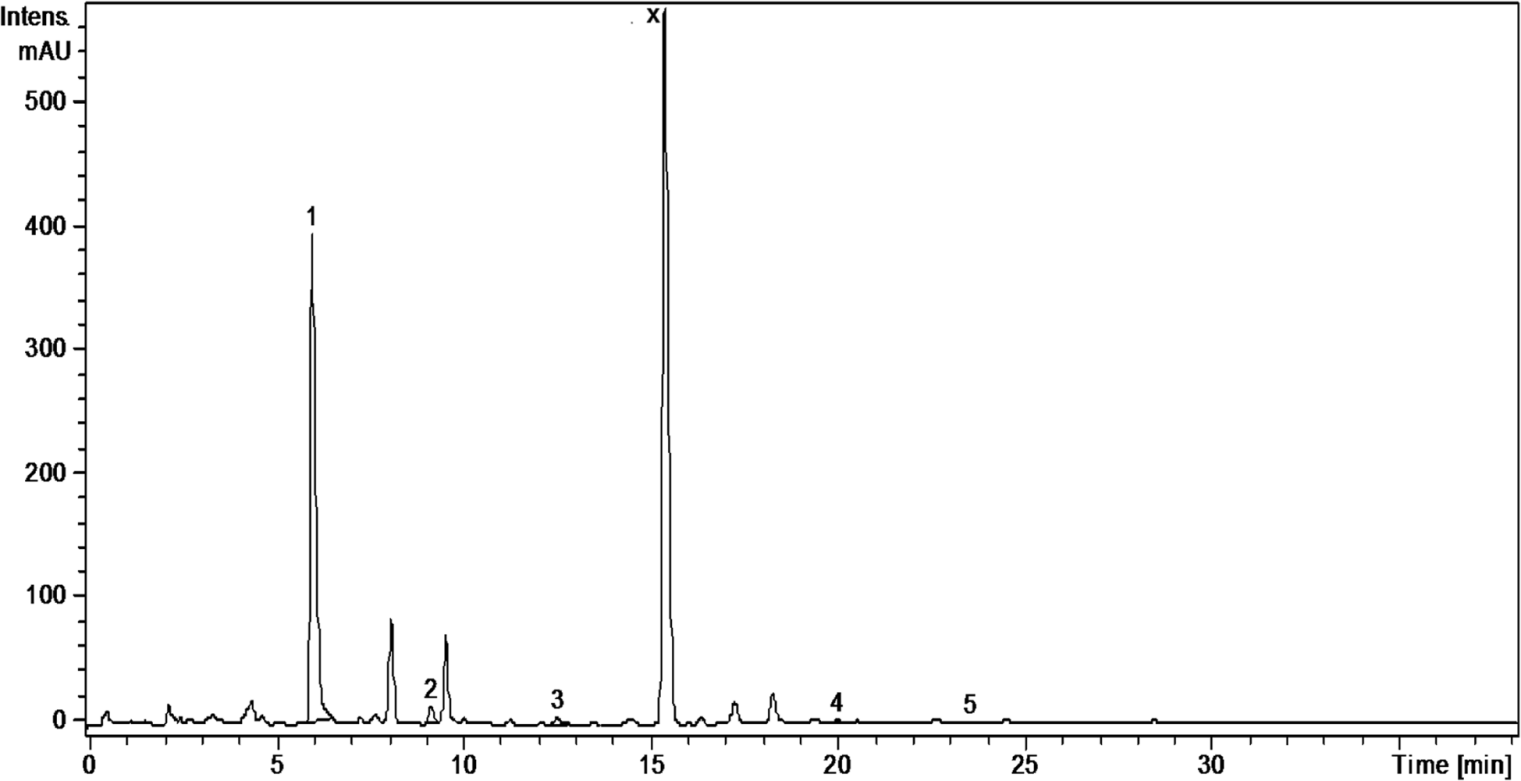
HPLC chromatograms of *G. nivalis* extract. *Notes*: The identified compounds: 1-chlorogenic acid; 2-*p*-coumaric acid; 3-ferulic acid; 4-isoquercitrin; 5-quercitrin

**Fig. 4:**
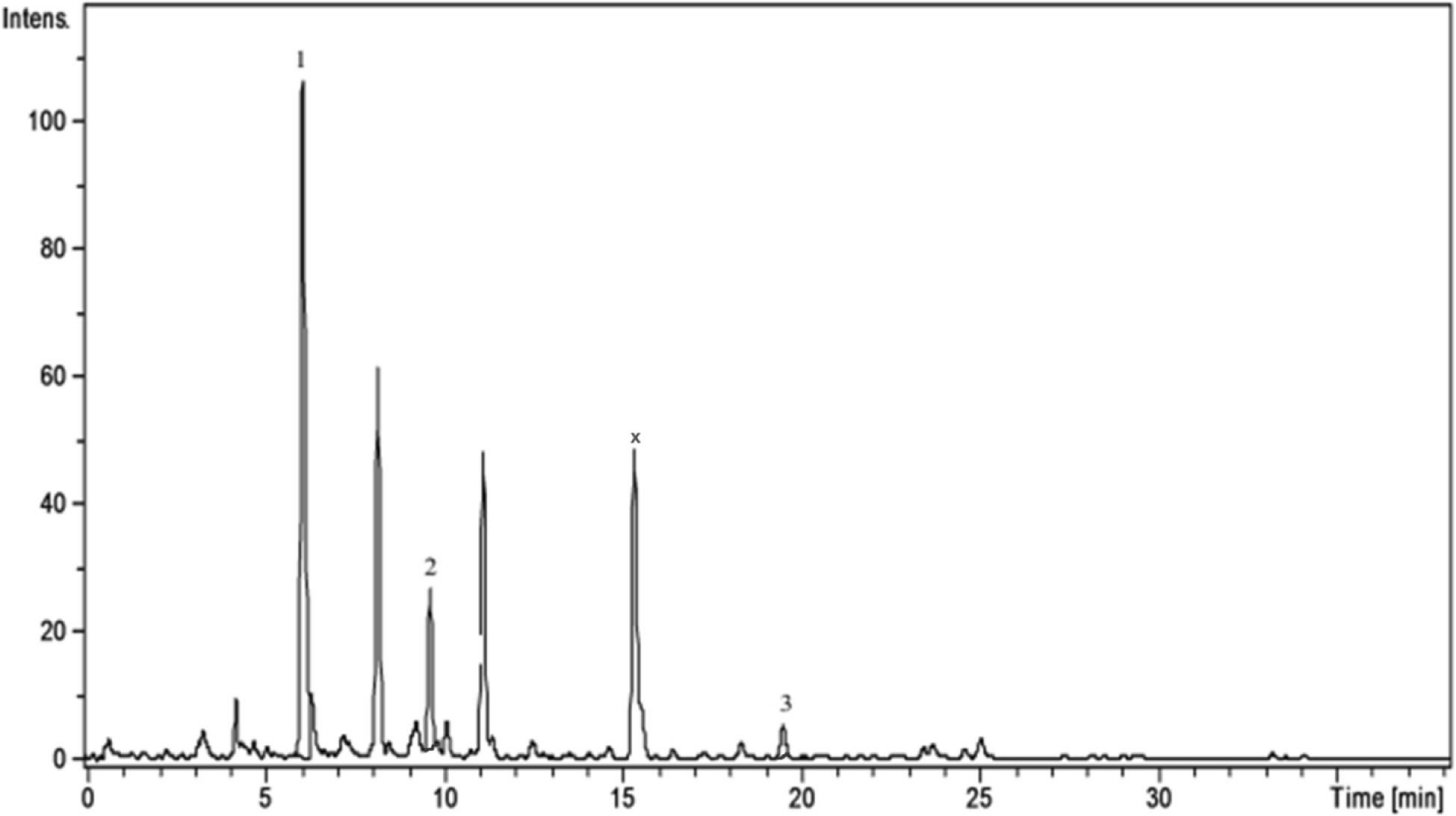
HPLC chromatograms of *L. vernum* extract. *Notes*: The identified compounds: 1-chlorogenic acid; 2-*p*-coumaric; 3-isoquercitrin

Analysis of the phenolic profile of the four Amaryllidaceae extracts was performed by a previously tested HPLC-MS method [15–19]. The identification of the compounds was based on their retention times, UV and MS spectra as compared to the standards. Thus, polyphenolic acids (e.g. gentisic, chlorogenic, *p*-coumaric, ferulic acids) and flavonoids (hyperoside, isoquercitrin, rutin, quercitrin, quercetin, kaempferol) were identified in the studied extracts using HPLC-MS analysis (Table 1, Figs. 1, 2, 3, and 4). Quantification of compounds was performed using an external standard method with 20 standard phenolic compounds (9 phenolic acids and 11 flavonoids).

**Table 1:** The polyphenolic compounds analyzed by HPLC-MS (μg/g plant material)

Compounds	[M-H]-m/z	Retention time (Rt), min	***N. pseudonarcissus***	***N. poeticus***	***G. nivalis***	***L. vernum***
neochlorogenic acid (y)	353	3.30 ± 0.00	NF	< 0.02	NF	NF
gentisic acid	179	3.69 ± 0.04	< 0.02	< 0.02	< 0.02	< 0.02
chlorogenic acid	353	6.43 ± 0.05	1425.56 ± 10.63	755.93 ± 4.06	2976.19 ± 12.80	1925.69 ± 6.31
cryptochlorogenic acid (z)	353	7.10 ± 0.00	NF	< 0.02	NF	NF
*p*-coumaric acid	163	9.48 ± 0.08	46.54 ± 0.45	32.70 ± 0.29	73.02 ± 0.07	270.51±
ferulic acid	193	12.8 ± 0.10	78.88 ± 1.11	25.29 ± 0.70	26.80 ± 0.19	< 0.02
quercetin-*O*-sophoroside or quercetin-3-*O*-galactosyl-(1 → 6)-galactoside	625	15.4 ± 0.00	< 0.02	< 0.02	< 0.02	< 0.02
hyperoside	463	18.60 ± 0.12	146.05 ± 1.94	NF	NF	< 0.02
isoquercitrin	463	20.29 ± 0.10	< 0.02	6.59 ± 0.10	25.08 ± 0.31	8.76 ± 0.19
rutin	609	20.76 ± 0.15	< 0.02	NF	NF	NF
quercitrin	447	23.64 ± 0.13	31.69 ± 0.10	9.26 ± 0.23	11.13 ± 0.06	< 0.02
quercetin	301	27.55 ± 0.15	< 0.02	NF	< 0.02	NF
kaempferol	285	32.48 ± 0.17	4.82 ± 0.07	NF	NF	NF

Values are the mean ± SD (*n* = 3). NF - not found, below limit of detection

### Total polyphenolic and flavonoidic contents

**Fig. 5:**
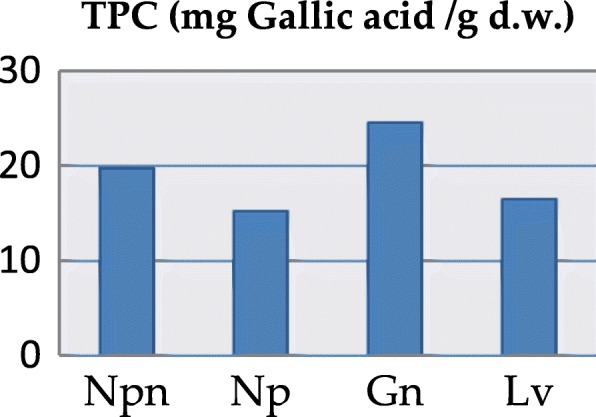
Total phenol content (TPC) of Amaryllidaceae species

**Fig. 6:**
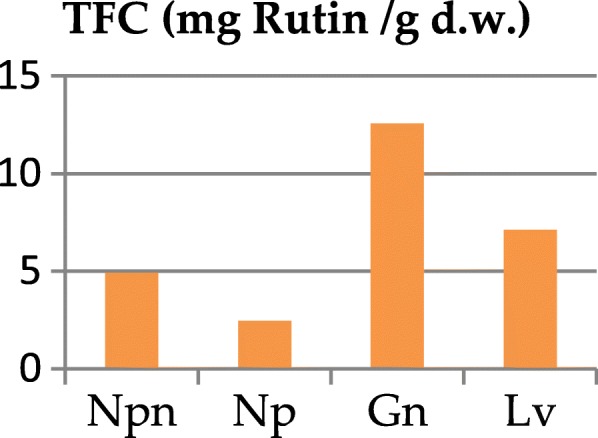
Total flavonoid content (TFC) of Amaryllidaceae species

The total content of polyphenols (TPC) and flavonoids (TFC) as well as the antioxidant activity of the four extracts revealed significant differences between the studied samples. The total polyphenolic content decreased in the following order: *G. nivalis* (24.57 mg/g) > *N. pseudonarcissus* (19.74 mg/g) > *L. vernum* (16.49 mg/g) > *N. poeticus* (15.25 mg/g) (*p* <  0.001), cf. Table 2, Fig. 5. The total flavonoidic content (Table 2, Fig. 6), using rutin as standard, decreased as follows: *G. nivalis* (12.56 mg/g) > *L. vernum* (7.10 mg/g) > *N. pseudonarcissus* (4.92 mg/g) > *N. poeticus* (2.47 mg/g).

**Table 2:** Total polyphenolic content (mg/g) and the results of antioxidant activities

Samples	TPC mg GAE/g	Flavonoids mg RE/g	DPPH IC_50_ μg Trolox/mL	FRAP μM Trolox /100 mL	HAPX (100)
*N. pseudonarcissus*	19.74 ± 0.25^a^	4.92 ± 0.17^c^	> 200	220 ± 10^e^	11 ± 4
*N. poeticus*	15.25 ± 0.06^b^	2.47 ± 0.12^c^	> 200	274 ± 5^e^	ND*
*G. nivalis*	24.57 ± 1.42	12.56 ± 1.43	139.88 ± 5.11^e^	725 ± 18^e^	35 ± 12
*L. vernum*	16.49 ± 0.50^b^	7.10 ± 0.09^d^	> 200	238 ± 7^e^	ND*
Trolox	–	–	11.20 ± 0.20	2073.91 ± 26.08	–

Notes: Each value is the mean ± SD of three independent measurements

*GAE* gallic acid equivalent, *RE* rutin equivalent, *Npn Narcissus pseudonarcissus*, *Np N. poeticus*, *Gn Galanthus nivalis*, *Lv* *Leucojum vernum*

^a^*p* < 0.05 (Gn versus Npn)

^b^*p* < 0.001 (Gn versus Np, Gn versus Lv)

^c^*p* < 0.001 (Gn versus Npn, Gn versus Np)

^d^*p* < 0.05 (Gn versus Lv)

^e^*p* < 0.001 (all samples versus Trolox); *ND – has not statistically significant effect

### Antioxidant activities evaluation

**Fig. 7:**
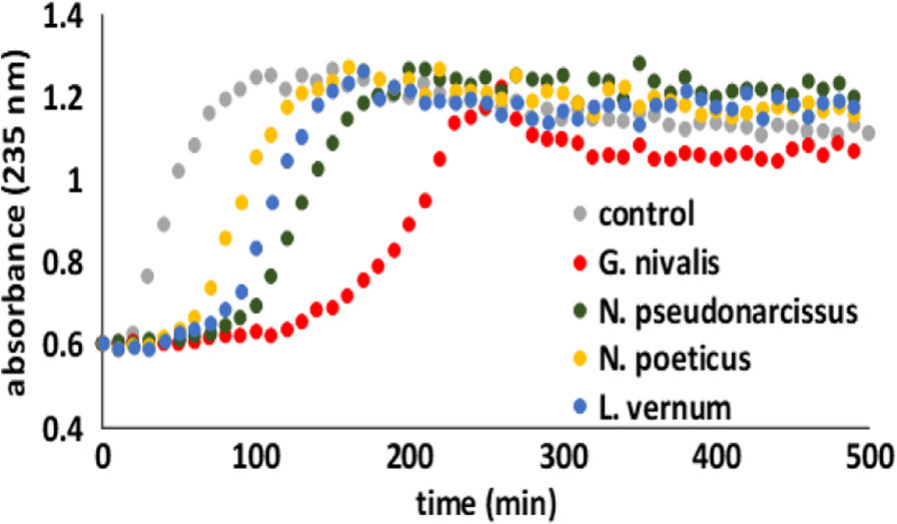
Liposome oxidation by cytochrome *c* in the presence of *G. nivalis, N. pseudonarcissus, N. poeticus* and *L. vernum* extracts, in phosphate buffer, pH 7, at room temperature

**Fig. 8:**
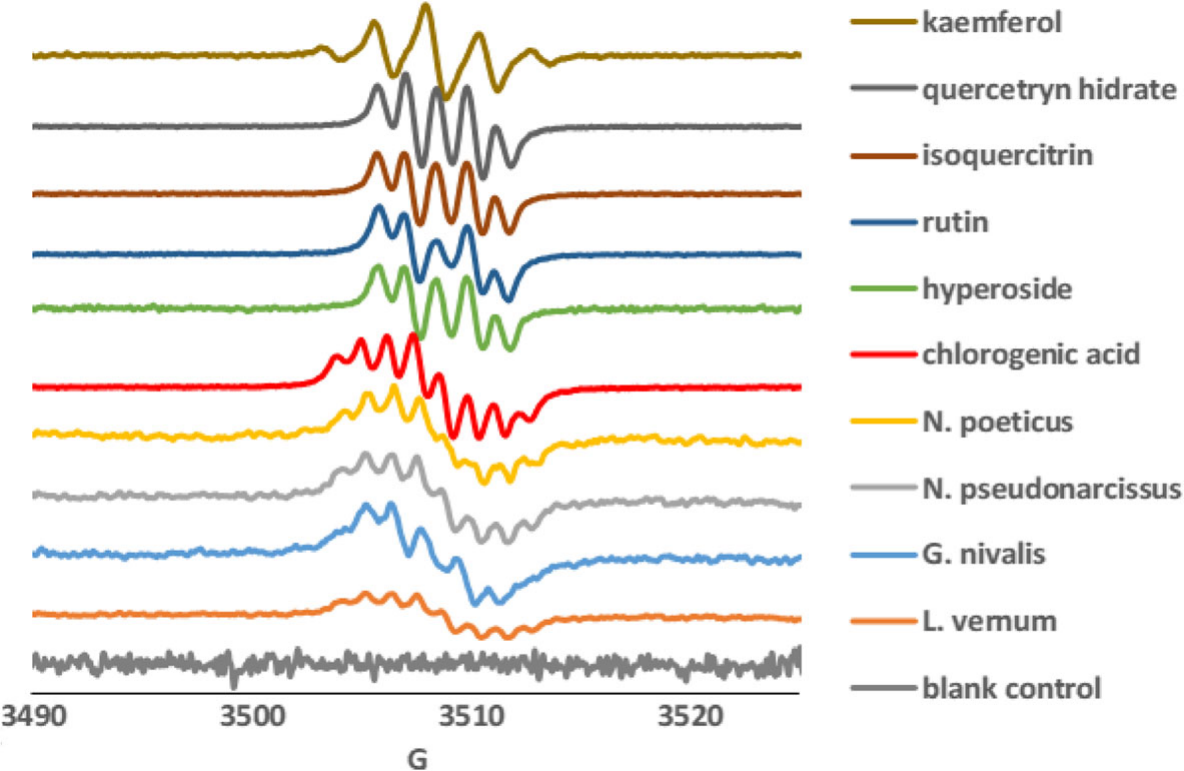
EPR spectra of Amaryllidaceae species and of references compounds, treated with NaOH, in ethanol 90%. The blank control sample contains only ethanol 90% and NaOH

The antioxidant potential was evaluated by several in vitro models, such as: DPPH•, FRAP bleaching system, hemoglobin ascorbate peroxidase activity inhibition (HAPX), inhibition of lipid peroxidation catalyzed by cytochrome *c*, and electron paramagnetic resonance (EPR) spectroscopy. The antioxidant activities quantitatively determined by the DPPH radical bleaching and FRAP methods, expressed as Trolox equivalents (Table 2), showed the same decreasing order of the activity: *G. nivalis* > *N. poeticus* > *L. vernum* > *N. pseudonarcissus.* For HAPX, and effect on the capacity to inhibit ferryl formation (methodology previously described [28–31]) was measurable only for *G. nivalis* and *N. pseudonarcissus* (given as percent of inhibition in Table 2). In terms of inhibiting the cytochrome *c* – induced lipid peroxidation, all extracts showed antioxidant capacity (Fig. 7). The EPR spectra detected 2 min after alkali treatment are shown in Fig. 8 and also reflect, qualitatively, the main polyphenolic components in the samples (to the extent to which they are prone to autooxidation under alkaline conditions).

### Determination of antimicrobial activity

Diameters of growth inhibition zones of ethanolic extracts from the *four Amaryllidaceae species* were compared with those of standards such as gentamicin for antibacterial activity and fluconazole and amphotericin B for antifungal activity. The *samples* showed various degrees of the inhibition against 4 bacterial strains and two fungus strains using the agar disk diffusion method (Table 3)*.* In terms of MIC, the same bacterial and fungal strains were used. The results in Table 4 showed that the MIC values varied from 19.53 to 625 mg/mL, for the 4 extracts (Table 4).

**Table 3:** Antimicrobial activity of the extracts

Samples	Diameter of inhibition zone (mm)					
	*S. enteritidis*	*E. coli*	*L. monocytogenes*	*S. aureus*	*C. albicans*	*A. brasiliensis*
*N. pseudo- narcissus*	6 ± 0.00^a^	6 ± 0.00^a^	12 ± 1.00^a^	14 ± 1.00^b^	18 ± 0.00^d^	16 ± 0.00^f^
*N. poeticus*	6 ± 0.00^a^	6 ± 0.50^a^	12 ± 0.00^a^	16 ± 2.00^c^	28 ± 0.50^e^	22 ± 0.00^g^
*G. nivalis*	6 ± 0.00^a^	6 ± 0.00^a^	10 ± 0.50^a^	18 ± 2.00^c^	6 ± 0.50^d^	16 ± 2.00^g^
*L. vernum*	6 ± 0.00^a^	6 ± 0.00^a^	10 ± 1.00^a^	22 ± 1.00^c^	6 ± 0.00^d^	6 ± 0.00^f^
Gentamicin	19 ± 1.00	18 ± 1.06	22 ± 0.50	18 ± 0.00	–	–
Fluconazole	**–**	–	–	–	25 ± 1.00	–
Amphotericin B	**–**	–	–	–	–	21 ± 0.00

*Notes:* The values represent the average of three determinations±SD. ^a^*p* < 0.001 (all sample versus Gentamicin); ^b^*p* < 0.05 (Npn versus Gentamicin); ^c^*p* > 0.05 (Np, Gn, Lv versus Gentamicin)

^b^*p* < 0.05 (Npn *versus* Gentamicin)

^c^*p* > 0.05 (Np, Gn, Lv *versus* Gentamicin)

^d^*p* < 0.001 (Npn, Gn, Lv versus Fluconazole)

^e^*p* < 0.05 (Np versus Fluconazole)

^f^*p* < 0.001 (Npn, Lv versus Amphotericin B)

^g^*p* < 0.05 (Np, Gn versus Amphotericin B)

Gentamicin (10 μg/disk), Fluconazole (25 μg/disk), Amphotericin B (10 μg/disk),) were used as positive controls

**Table 4:** Minimum inhibitory concentration (MIC) values

Samples	MICs (μg/mL)					
	*S. enteritidis*	*E. coli*	*L. monocytogenes*	*S. aureus*	*C. albicans*	*A. brasiliensis*
*N. pseudo- narcissus*	625	2500	156.25	78.13	39.06	78.13
*N. poeticus*	625	2500	78.13	78.13	19.53	19.53
*G. nivalis*	625	2500	312.50	19.53	1250	78.13
*L. vernum*	625	2500	625	19.53	2500	2500
70% Ethanol	625	2500	1250	625	2500	2500

## Discussions

Polyphenolic compounds were identified in the studied extracts, using HPLC-MS analysis (Table 1, Figs. 1, 2, 3, and 4); some of them were reported here for the first time (gentisic acids, *p*-coumaric acids, hyperoside, quercetrin). Chlorogenic acid dominated the phenolic acid profiles of all samples, its concentration decreasing in the following order: *G. nivalis* > *L. vernum* > *N. pseudonarcissus* > *N. poeticus*. Ferulic and *p*-coumaric acids were found in all studied samples. The *L. vernum* extract was the richest in *p*-coumaric acid (270.51 μg/g), while the largest amount of ferulic acid was determined in *N. pseudonarcissus* (78.88 μg/g). Gentisic acid was detected only in traces. Bulgarian authors reported the presence of chlorogenic and ferulic acid in aerial parts of *G. nivalis* and *L. aestivum* [14]. In *G. elwesii* extracts, *p*-hydroxybenzoic acid was reported as the major constituent, followed by vanillic and ferulic acids [11]. In terms of flavonoids, there are qualitative and quantitative differences between the species examined in the present study. In the *N. pseudonarcissus* extract, 4 glycosides (hyperoside, isoquercitrin, rutin and quercitrin) and 2 aglycones of flavonoids (quercetin and kaempferol) were determined. Hyperoside was quantified in a large amount (146.05 μg/g), followed by quercitrin (31.69 μg/g). Isoquercitrin, rutin and quercetin were found in too low concentrations to be quantified (< 0.02). Isoquercitrin was quantified in *N. poeticus* (6.59 μg/g), *G. nivalis* (25.08 μg/g) and *L. vernum* (8.76 μg/g). Quercitrin was determined in *N. pseudonarcissus* (31.69 μg/g), *N. poeticus* (9.26 μg/g) and *G. nivalis* (11.13 μg/g). Others have identified flavonoids (quercetin, kaempferol etc.) and chlorogenic acid in perianths and coronas of Chinese *Narcissus* cultivars [13]. Elsewhere, quercetin-3-*O*-sophoroside and kaempferol-3-*O*-sophoroside were found in the tinctures of various *Galanthus* sp., [4]. In the present study, the HPLC chromatogram of the *G. nivalis* extract showed a major peak at Rt = 15.4 min., for which no standards were available. In the fragmentation, this compound (with m/z = 625; marked x) has a main fragment with m/z 300, which could be a quercetin moiety and a fragment with m/z = 325, possible two hexoses (glucose, galactose) (Fig. 3). This compound may also be present in other samples, such as *N. pseudonarcissus* extract, *N. poeticus* extract and *L. vernum* extract (Figs. 1, 2 and 4). Comparing with literature data, the peak could correspond to: quercetin-*O*-sophoroside or quercetin-3-*O*-galactosyl-(1 → 6)-galactoside (molecular weight = 626) [4]. The high level of this flavonoidic compound can explain the large amount of total flavonoids in *G. nivalis*. The HPLC chromatogram of the *N. poeticus* extract showed two peaks at Rt = 3.30 and 7.10 min. For which no standards were available. The compounds marked with y and z ([M-H]^−^ions; m/z 353) are presumed to be a type of caffeoylquinic acid: 4-caffeoyl-quinic acid and/or 5-caffeoyl-quinic acid, as isomers of chlorogenic acid, in accordance with literature data [34]. In addition, the chlorogenic acid showed a molecular ion peak at (m/z 353) and a fragmentation ion that corresponds to the deprotonated quinic acid (m/z 191) [35]. The evaluation of negative electrospray MS spectra shows that compound y has m/z value = 353 and is single-charged (the isotopic forms are at 1 amu difference), so the molecular mass is 354. In the fragmentation, the compound y has a main fragment with m/z 191, which could be a quinic acid moiety and a fragment with m/z = 179 which could be the caffeic acid. MS spectra showed that compound z has the molecular weight = 353 (M-H+). In the fragmentation, compound z has the main fragments with m/z values: 173 - resulting from the subsequent loss of water (− 18 Da) [quinic acid–H–H_2_O]^−^; 191 (quinic acid); 135; 155. We propose a quinic acid ester structure (molecular weight of 354) for compounds y and z, based on comparison with literature data: neochlorogenic acid (5-*O*-caffeoylquinic acid) or/and cryptochlorogenic acid (4-O-caffeoylquinic acid) [34]**.**

Because of their content in chlorogenic acid, the herein examined extracts could be important sources of these active principles (especially *G. nivalis*), with a great role in preventing various diseases associated with oxidative stress: cancer, cardiovascular, aging, neurodegenerative diseases (Alzheimer’s disease) etc. [36, 37]. Also, the *N. pseudonarcissus* extract could be exploited for its hyperoside content, with well-known properties, such as antiviral, antioxidative, antiapoptotic or anti-inflammatory [38].

The total content of polyphenols is considered high if the value is between 12 and 20 mg GAE/g dried plant product [10, 39–41]. In line with this observation, the phenolic content was high for all samples (> 15 mg GAE/g) (Table 2, Fig. 5). In the case of Hungarian *L. vernum* leaves, a higher total polyphenolic content was noted (22.71 mg GAE/g) [10]. On the other hand, for the flowers of *G. nivalis* (from Dobrogea County, Romania) a very low content was reported (0.08–0.13 mg GAE/g dry weight) [42]. In terms of total flavonoid content, the extract of *G. nivalis* was the richest. A one-way ANOVA test applied on the values flavonoidic content (Table 3) showed highly significant differences between *G. nivalis* and both samples of *Narcissu*s (*p* <  0.001) and significant differences between *G. nivalis* and *L. vernum* (*p* <  0.05).

The antioxidant activity of the extracts measured by DPPH and FRAP was effective in the order *G. nivalis* > *N. poeticus* > *L. vernum* > *N. pseudonarcissus*. The highest radical scavenging activity was shown by *G. nivalis* (139.88 μg/mL). The other extracts did not show antioxidant activity (> 200 μg/mL). The antioxidant activity is probably due to the presence of polyphenols, such as flavonoids, caffeic acid derivatives, in the alcoholic extracts [9]. The *G. nivalis* extract showed 3 times higher antioxidant activity compared to three other extracts (*p* < < 0.001). This activity was statistically significantly inferior to Trolox used as reference antioxidant (*p* < << 0.001). In good agreement with the trends discussed above, the HAPX effect was measurable only for *G. nivalis* and *N. pseudonarcissus*. For the other samples, no significant results were obtained using 200-fold diluted extracts; at higher concentrations, the reaction could not be monitored due to the interference between the spectra of hemoglobin and extracts.

The inhibition of lipid peroxidation catalyzed by cytochrome *c* is based on the interaction of antioxidants with ferryl generated in this case in cytochrome *c* and/or with free radicals involved in cytochrome-induced autooxidation of lipids [31]. *G nivalis* exhibits the best antioxidant capacity, reflected in a longer induction time (100 min.) than that observed for *N. poeticus* (40 min.), *L. vernum* (50 min.) and *N. pseudonarcissus* (60 min.). Regarding *G. nivalis* displaying the highest antioxidant capacity of this group, these results are in agreement with DPPH, FRAP and HAPX experiments.

The EPR spectra detected 2 min after alkali treatment of the extracts have a hyperfine structure at room temperature which for *N. poeticus* and *N. pseudonarcissus* is very similar with that of chlorogenic acid. While the *G. nivalis* extract contains the highest amount of chlorogenic acid, the EPR signal is not dominated by this species but rather appears to be a mixture of isoquercitrin, quercitrin and chlorogenic acid free radical signals. The spectrum of *L. vernum* is much weaker and has an incompletely defined line shape most probably due to a mixture of isoquercitrin, rutin and quercetrin which are found in a small amount. The free radical signals in all four samples were stable in time and may be attributed to the formation of semiquinone anion radicals within the polyphenols [29, 30]. According to the HPLC-MS analysis, all extracts contain notable amounts of *p*-coumaric acid; however this compound did not yield EPR signals under the conditions described herein.

With regard to antibacterial activity, all extracts demonstrated anti-staphylococcal activity on *S. aureus*: *G. nivalis* extract exhibited antibacterial action similar to gentamicin (inhibition diameter - 18 mm) and *L. vernum* extract demonstrated a higher activity than the reference antibiotic (inhibition diameter - 22 mm). This activity could be in accordance with the high content of phenolic acids (chlorogenic acid and *p*-coumaric acid) in the 2 extracts [43, 44]. The two *Narcissus* extracts showed moderate anti-staphylococcal activity (inhibition diameter between 14 and 16 mm). All the samples showed less activity on *S. enterides, E. coli* and *L. monocytogenes* (*p* <  0.001) compared with gentamicin used as reference antibiotic. The activity on *S. aureus* was almost similar with gentamicin for *N. pseudonarcissus*, *N. poeticus* and *G. nivalis* (*p* <  0.01) and slightly superior for *L. vernum* (*p* <  0.005). The extracts tested on gram-negative bacteria: *S. enteritidis* and *E. coli* have been shown to be inactive (diameter of the inhibition zone - 6 mm, as well as the negative control). In terms of antifungal activity, the extract of *N. pseudonarcissus* showed moderate antifungal activity on the two tested fungus (inhibition diameter between 16 and 18 mm), whereas *N. poeticus* extract showed a strong effect (*C. albicans:* inhibition diameter - 28 mm; *A. brasiliensis:* 22 mm), even greater than fluconazole (*p* <  0.01) and amphotericin B (*p* = 0.02). Some Czech alkaloidal extracts of *Narcissus sp.* (*N. poeticus* var. *recurvus*, *N. jonquilla*) and *Leucojum aestivum* presented a strong anti-yeast activity [6]. *Narcissus sp.* aqueous extract showed antibacterial action that was explained by the occurrence of polyphenolic compounds in the extract [6]. Regarding the action on *L. monocytogenes*, the activity of the four extracts was weak to moderate (inhibition diameter between 10 and 15 mm). In terms of antifungal activity, *L. vernum* extract was inactive on the two tested fungi (*Candida albicans* and *Aspergillus brasiliensis*, diameter = 6 mm), while *G. nivalis* extract was inactive on *C. albicans.* Our results suggest that the studied plant extracts showed antibacterial activity against gram positive bacterial strains and antifungal effect. Thus, *G. nivalis* and *L. vernum* extracts have demonstrated a good anti-staphylococcal activity, while the *N. poeticus* extract has shown a remarkable anti-*Candida* effect. Concerning the minimal inhibitory concentration, the lowest MIC values (19.53 μg/mL) were obtained for the *G. nivalis* and *L. vernum* extracts against *S. aureus* and also for *N. poeticus* on fungi (*C. albicans* and *A. brasiliensis*). The results are consistent with those obtained by the diffusion method. Based on these in vitro results, the polyphenolic-rich tested extracts could be used for further studies in order to propose new antimicrobial agents.

## Conclusions

The phenolic profile, antimicrobial and antioxidant activity of some Romanian Amaryllidaceae species (*G. nivalis, N. pseudonarcissus, N. poeticus* and *L. vernum*) were analyzed. The results indicated that the aerial parts of these species contain considerable amounts of polyphenols, with chlorogenic acid as major compound. The *G. nivalis* and *L. vernum* extracts have demonstrated a very good anti-staphylococcal activity, while the *N. poeticus* extract has shown a remarkable anti-*Candida albicans* effect. These species of Amaryllidaceae family are important not only for the presence of alkaloids, but also for their content in polyphenols in the aerial parts, with a good antimicrobial activity.

The results of our research complete the pharmacognostical characterization of some Amaryllidaceae species with new data and recommend them in further studies on finding alternative sources as anti-staphylococcal and antifungal agents.

## Abbreviations

d.w.: Dry weight
DPPH: 2,2-diphenyl-1-picrylhydrazyl
FRAP: Ferric reducing antioxidant power
GAE: Gallic acid equivalent
Gn: *Galanthus nivalis*
HAPX: Hemoglobin ascorbate peroxidase activity inhibition
Lv: *Leucojum vernum*
MICs: Minimum inhibitory concentrations
Np: *N. poeticus*
Npn: *Narcissus pseudonarcissus*
RE: Rutin equivalent
TFC: Total flavonoid content
TPC: Total phenol content
TPTZ: 2,4,6-tri(2-pyridyl)-1,3,5-triazine
*Trolox*: 6-hydroxy-2,5,7,8-tetramethylchroman-2-carboxylic acid
